# The Description of the Extremophile *Reticulonema bolivianum* gen. et sp. nov. (Microcoleaceae, Cyanobacteria) and the Review of the Phylogenetic Status of the Genus *Capilliphycus* Based on the 16S rRNA Gene

**DOI:** 10.3390/plants14030310

**Published:** 2025-01-21

**Authors:** Guilherme Scotta Hentschke, Claudia Hoepfner, Daniel Guzmán, Vitor M. Vasconcelos

**Affiliations:** 1 Interdisciplinary Centre of Marine and Environmental Research of the University of Porto, Terminal de Cruzeiros de Leixões, Av. General Norton de Matos s/n, 4450-208 Matosinhos, Portugal; 2 Center of Biotechnology, Faculty of Sciences and Technology, Universidad Mayor de San Simon, Cochabamba 2500, Bolivia; c.hoepfner@umss.edu (C.H.); ma.guzman@umss.edu (D.G.); 3 GEMA Center for Genomics, Ecology and Environment, Faculty of Interdisciplinary Studies, Universidad Mayor, Huechuraba 8580745, Chile; 4 Department of Biology, Faculty of Sciences, University of Porto, Rua do Campo Alegre, 4069-007 Porto, Portugal; vmvascon@fc.up.pt

**Keywords:** biodiversity, extreme environments, new genus, new species

## Abstract

This article describes a new genus and species of cyanobacteria isolated from Laguna Pastos Grandes in the Bolivian Altiplano. This discovery marks the first described species of this phylum from this extreme environment. Additionally, the phylogenetic status of the genus *Capilliphycus* is reassessed. The analyses are based on 16S rRNA gene maximum likelihood and Bayesian phylogenies, identity (p-distance), morphology and habitat comparisons. The new genus is a *Lyngbya*-like type from a mat at the margin of a brackish–alkaline lake with borax. It is phylogenetically close to *Dapis*, *Tenebriella* and *Okeania*, but compared to these genera, the maximum 16S rRNA gene identity values reached only 90.9%, 91.1% and 92.5%, respectively. The genus *Capilliphycus* was found to be polyphyletic. The type species *Capilliphycus salinus* is clustered with *C. guerandensis*. However, “*C. tropicalis*” and “*C. flaviceps*” form a distinct clade, distant from the *Capilliphycus* type species. Between the two “*Capilliphycus*” clades, *Sirenicapillaria* and *Limnoraphis* are found.

## 1. Introduction

The Bolivian Altiplano is a high plateau formed during the uplift of the Andes mountain range. Its extension reaches an approximate area of 200,000 km^2^ that hosts freshwater and saltwater lakes. Additionally, it harbors salt flats surrounded by mountains that cover much of its surface. All salt flats in the Bolivian Altiplano are at elevations above 3600 m above sea level (masl), which makes them extreme environments subjected to conditions of extreme temperature and radiation, among others. The climate in the Altiplano is considered to be arid to semi-arid, with annual rainfall ranging from 800 to 1000 mm in the north to less than 100 mm per year in the Uyuni salt flat region. Similarly, this region experiences highly fluctuating temperatures, varying from −20 °C during winter nights to 20 °C during summer days. Additionally, due to its elevation, ultraviolet (UV-A, UV-B) radiation levels in the region are considered very high [1,2,3].

In the southern region of Bolivia, commonly known as “Los Lípez”, lies Laguna Pastos Grandes, which is one of the largest basins in this part of the Bolivian Altiplano. Its total area is approximately 120 km^2^, and it is located at an elevation of 4450 masl. The western region of the lagoon is characterized by harboring a salt flat composed mainly of sodium chloride, sodium sulfate, and calcium sulfate, along with the presence of a carbonate crust. The lagoon is situated in a caldera, and the entire region in considered an active volcanic zone. As a result, part of the lagoon is fed by thermal springs with temperatures ranging from 20 to 75 °C [4,5].

The caldera has been dated to 2.89 ± 0.01 Mega-annum by 40/39 Argon dating of sanidine, with a geological origin explained by two consecutive ignimbrite eruptions. The eastern flank of the lagoon represents a well-preserved caldera wall, while the western flank is crossed by several dacitic stratovolcanoes from the Pliocene [6,7].

Studies on the microbiome of Laguna Pastos Grandes have mainly focused on ponds fed by thermal springs, initially identifying the presence of algae and diatoms [4]. Subsequently, diatoms such as *Surirella chilensi* Janish, *Surirella sella* Hustedt, *Navicula* sp., and *Amphora boliviana* Patrick were identified. Microbial mats, possibly formed by cyanobacteria, were also observed [5].

Subsequent studies analyzed the presence and formation of microbial mats and microbialites in the lagoon. The microbial presence is mainly associated with thermal spring systems, where green microbial mats, black microbial mats, and diatoms are primarily found. Green mats are typically subaqueous, capable of forming dense mats a few centimeters thick, with cyanobacterial filaments up to five centimeters long. It is believed that these microbial mats, present in pools formed by thermal springs, may influence carbonate precipitation and pisolith formation. Along the course of the thermal springs, microbialites of different shapes are also observed: ledge, mushroom-like, cerebroid, snake-like, and others [6,7,8].

Recent studies have expanded on this knowledge, showing that among eukaryotes, diatoms and crustaceans have the highest relative abundance in water samples. In the salt flat, archaea belonging to the Halobacteriales, and cyanobacteria of subsection III are the most abundant, with unclassified species of *Microcoleus* Gomont found among the Cyanobacteria [9].

Many of these extremophiles exhibit cryptic diversity, where organisms may appear morphologically identical but are separated by substantial genetic distances. The application of 16S rRNA gene phylogenetic analysis for Cyanobacteria classification [10,11] and the formal recognition of Cyanobacteria by the International Code of Nomenclature of Prokaryotes (ICNP) and the International Code of Nomenclature for algae, fungi, and plants (ICN) has brought about significant taxonomic revisions within the group. Since 2000, molecular analyses have led to the description of 1073 new cyanobacterial taxa [12], and numerous species have been renamed or reassigned to different genera based on phylogenetic evidence accumulated over time.

Among these newly described species, many are morphologically indistinguishable from known taxa, classified as “cryptic”. Kastovsky [12] notes that nearly a quarter of cyanobacterial species described since 2000 fall under this classification, underscoring the impact of molecular techniques on cyanobacterial taxonomy. The recognition of cryptic species and even “cryptogenera” has emerged as a crucial outcome of this molecular era, with such studies becoming essential to advancing our understanding of cyanobacterial biodiversity.

While ongoing discovery of new taxa continues to enrich our knowledge of Cyanobacteria, revisiting and refining the taxonomy of already-described genera has become equally vital. This effort is particularly important for resolving polyphyletic groups and refining higher-level classifications. One such revision is the separation of the genus *Capilliphycus* Caires et al. from *Lyngbya* Gomont, a clear example of how re-evaluating cryptic diversity can lead to significant changes in the understanding of cyanobacterial phylogeny [13,14].

In light of these advancements, the present study describes a new genus and species of cyanobacteria isolated from Laguna Pastos Grandes, sampled during a collection trip in 2023. This discovery marks the first described species of this phylum from this environment, thus preserving the great microbial diversity that remains to be discovered in the lagoon. Additionally, our analyses permitted the reassessment of the phylogenetic status of the Microcoleaceae genus *Capilliphycus*.

## 2. Results

**Figure 1:**
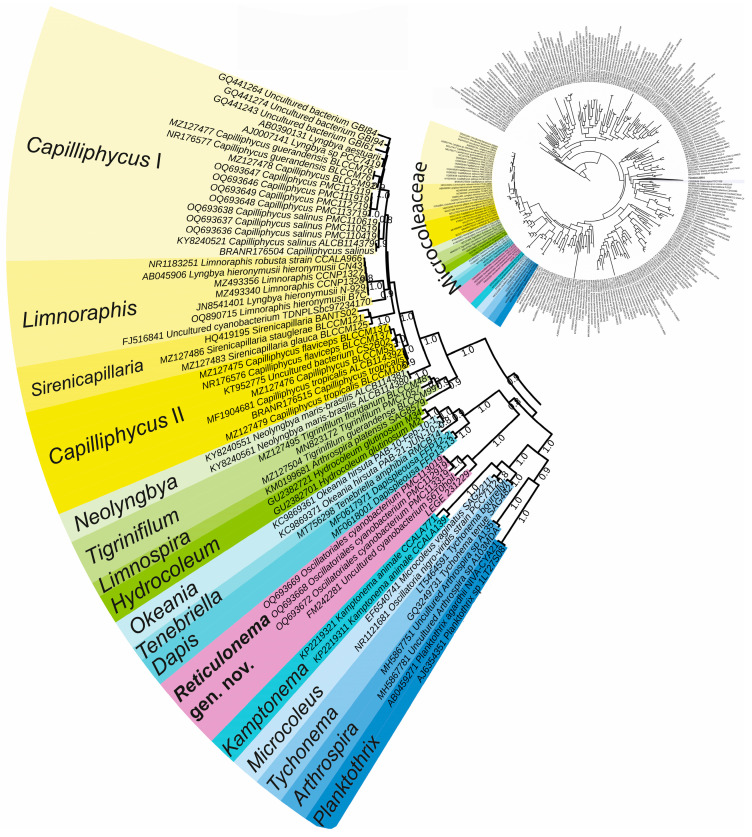
First-round analysis. 16S rRNA gene phylogenetic analysis of the studied strains and reference strains of Cyanobacteria. The Microcoleaceae genera are highlighted in different colors.

In the first-round phylogenetic analysis (Figure 1), the Microcoleaceae family proved to be monophyletic with strong statistical support (maximum likelihood (ML) = 90). Within this family, our isolate LEGE 231229 was positioned within a distinct clade alongside unidentified “Oscillatoriales” sequences. This clade presented strong phylogenetic support (ML = 100) and was a sister (ML = 80) to *Dapis*, *Okeania*, *Hydrocoleum*, and *Tenebriela*. This finding indicated that LEGE 231229 cannot be assigned to any Microcoleaceae from already-described genera.

In this phylogeny, we also found that the *Capilliphycus* species *Capilliphycus salinus* Caires et al. (type), *C. tropicalis* Caires et al., *C. flaviceps* Berthold & Laughinghouse, and *C. guerandensis* Lefler et al. were placed within a cluster alongside *Limnospira* Nowicka-Krawczyk et al., *Tigrinifilum* Berthold et al., *Neolyngbya* Caires et al., *Sirenicapillaria* Berthold et al., and *Limnoraphis* Komárek et al.

The topology of the tree indicated that the type *C. salinus* was clustered (ML = 80) with *C. guerandensis*, making the genus coherent. We labeled this clade *Capilliphycus* I. Nevertheless, the species *C. tropicalis* and *C. flaviceps* were in a distinct clade (“*Capilliphycus*” II, ML = 100), distant to the type species, indicating that the genus was polyphyletic. Between the two *Capilliphycus* sensu lato clades, *Sirenicapillaria* and *Limnoraphis* were placed, and the phylogenetic relationships among all these genera exhibited strong phylogenetic support (Figure 1).

**Figure 2:**
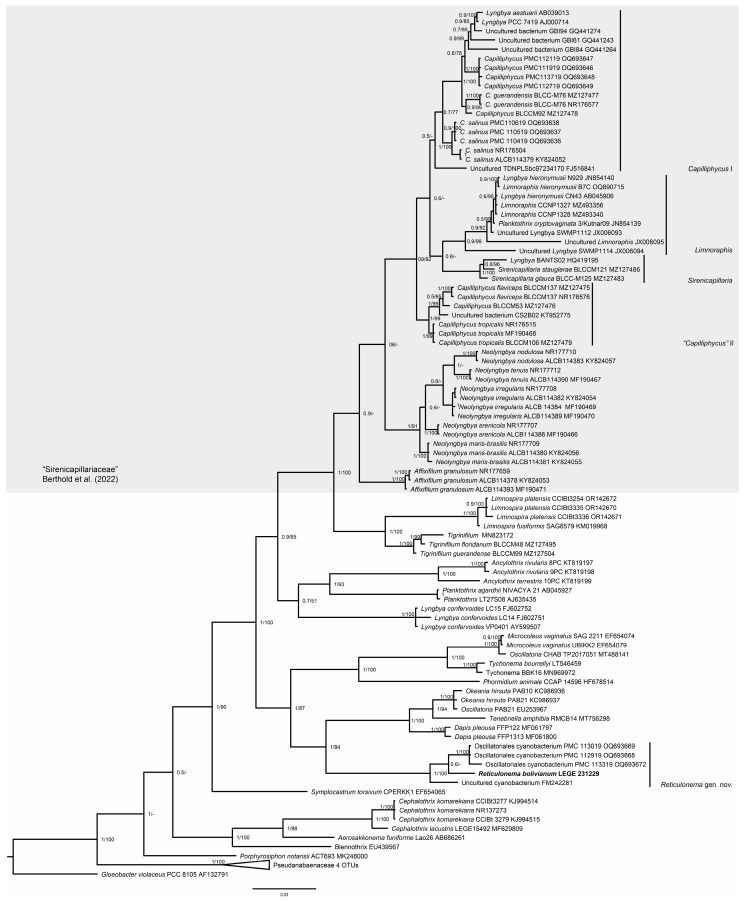
Second-round analysis. Microcoleaceae 16S rRNA gene Bayesian Inference phylogenetic tree. Posterior probabilities and bootstrap supports are indicated at the nodes. The grey shade indicates the former “Sirenicapillariaceae” genera. Berthold et al. (2022) [15].

In the second-round analyses, the phylogenetic BI and ML trees (Figure 2) exhibited similar backbone topology and robust phylogenetic support. The Pseudanabaenaceae clade (Bayesian Inference (BI) = 1, ML = 100) was situated at the base of both phylogenies and was the sister group of the Microcoleaceae cluster (BI = 1, ML = 100). At the base of the Microcoleaceae, *Porphyrosiphon* Gomont and *Blennothrix* Anagnostidis & Komárek were placed, followed by *Aerosakkonema* Thu & Watanabe and *Cephalothrix* Malone et al. (BI = 1, ML = 97). The next derivation in both trees was *Symplocastrum* Kirchner, followed by a cluster containing *Tychonema* Anagnostidis & Komárek, *Microcoleus* Gomont, *Dapis*, *Tenebriella*, and *Okeania* (BI = 0.99 = ML = 93). Within this cluster, we also found a clade formed by our isolate LEGE 231229 alongside four unidentified “Oscillatoriales” sequences. This clade was distinct from the other Microcoleaceae genera, was monophyletic, and exhibited strong phylogenetic support (BI = 1, ML = 100). This is in agreement with the first-round phylogeny and indicates that this clade should be described as a new cyanobacterial genus.

The 16S rRNA gene identity analysis corroborated with the description of LEGE 231229 as a distinct genus (Table 1). The intraclade identity values within the LEGE 231229 clade ranged from 97.8 to 100%. This clade also includes the strains PMC 1129.19, 1130.19, and 1133.19 and the uncultured bacterium (FM242281). When compared to *Dapis*, *Tenebriella*, and *Okeania*, the phylogenetically closest related genera, the maximum identity values reached only 90.9%, 91.1%, and 92.5%, respectively.

Morphologically, LEGE 231229 is a *Lyngbya*-like type, with motile trichomes attenuated toward the ends and without mucilaginous sheaths. The terminal cells are mainly conical and the others are discoid with aerotopes (Table 2). LEGE 231229 is similar to its phylogenetically closest relatives but differ from them in many aspects. For instance, *Dapis* trichomes are not attenuated toward the ends and exhibit mucilaginous sheaths; moreover, the aerotopes are lacking in this genus. LEGE 231229 also differs from *Tenebriella* and *Okeania* by the lack of aerotopes in the latter two genera. Regarding habitats, it was also possible to differentiate LEGE 231229 from the closest phylogenetic relatives. Among this cluster, LEGE 231229 was the only genus present that is related to brackish and alkaline environments. *Dapis, Tenebriella*, and *Okeania* are primarily marine.

Based on the phylogenetic, 16S rRNA gene identity analysis, morphological, and habitats comparisons, LEGE 231229 proved to be a distinct cyanoabacterial genus.

The second-round phylogenies (Figure 2) also revealed that the “Sirenicapillariaceae” cluster presented strong phylogenetic support (BI = 1, ML = 100). All the genera within this cluster proved to be monophyletic and coherent, with the exception of *Capilliphycus*, which was polyphyletic. These results corroborated the first-round analysis’ findings.

The type species *C. salinus* (*Capilliphycus* I clade) clustered with *C. guerandensis*, “*Lyngbya. aestuarii*”, and the strain *Capilliphycus* PMC, showing strong phylogenetic support (BI = 1, ML = 100) (Figure 2). This clustering indicated that the genus was distinct from other Microcoleaceae genera. However, “*C. tropicalis*” and “*C. flaviceps*” (“*Capilliphycus*” II) formed a separate clade, also with strong phylogenetic support (BI = 1, ML = 98), distant from the *Capilliphycus*-type species. As in the first-round analysis, both *Capilliphycus* clades were separated from each other by *Sirenicapillaria* and *Limnoraphis*.

The 16S rRNA gene identity among “Sirenicapillariaceae” genera is summarized in Table 3. The data show that the intrageneric identity values ranged from a minimum of 96.4% (*Capilliphycus* I) to a maximum of 100% (*Capilliphycus* I, “*Capilliphycus*” II, *Sirenicapillaria*, *Limnospira*, *Neolyngbya*, *Limnoraphis*, and *Affixifilum)*. The mean identity within “Sirenicapillariaceae” was 96.4% (±1.5). Comparing “Sirenicapillariaceae” genera among them, the mean identity values varied from a minimum of 94.7% (±1.1) to a maximum 96.2% (±1.1). However, some genera shared high identity values (above 95%), such when comparing *Limnoraphis* to *Sirenicapillaria*, which shared 96.5–97.3% of 16S rRNA gene identity. *Limnoraphis* also presented high identity with *Capilliphycus* I and “*Capilliphycus*” II clades, reaching 98.3% and 98.1%, respectively. Moreover, the identity between *Limnoraphis* and *Limnospira*, a non-*Lyngbya*-like genus with typical coiled trichomes, reached 95.5%.

*Sirenicapillaria* also shared high identity with both *Capilliphycus* I and II clades, reaching 97% (Table 3). Comparing the 16S rRNA gene identities among *Capilliphycus* I and “*Capilliphycus*” II clades, the values ranged between 95.6% and 98.4%, although they were phylogenetically separated from each other by *Sirenicapillaria* and *Limnoraphis*.

The morphological comparisons among *Capilliphycus* I, “*Capilliphycus*” II, and the phylogenetically and morphologically related genera are summarized in Table 4. Based on the morphological characters analyzed, it was not possible to establish an apomorphy for each of these genera. It was only possible to differentiate “*Capilliphycus*” II clade, *Capilliphycus* I, *Neolyngbya*, and *Affixifilum* from *Lyngbya* by the presence of aerotopes, which are lacking in *Lyngbya*. It was also possible to differentiate all genera from *Limnoraphis* when comparing habitats. *Limnoraphis* is the only genus that is typical from freshwater environments. It was not possible to distinguish the cryptic genera *Tigrinifilum, Affixifilum, Neolyngbya,* and *Capilliphycus* sensu lato by morphological traits.

*Description of the new taxon*

*Reticulonema bolivianum* G. S. Hentschke gen. et sp. nov.

**Figure 3:**
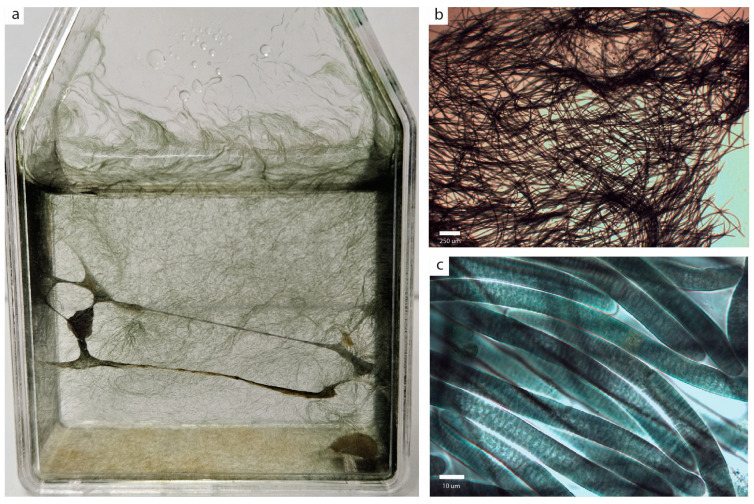
*Reticulonema bolivianum* gen. et sp. nov. (**a**) Macroscopic thallus growing in Z8 culture medium. (**b**) General aspects of entangled trichomes. (**c**) Detail of trichomes in colony.

**Figure 4:**
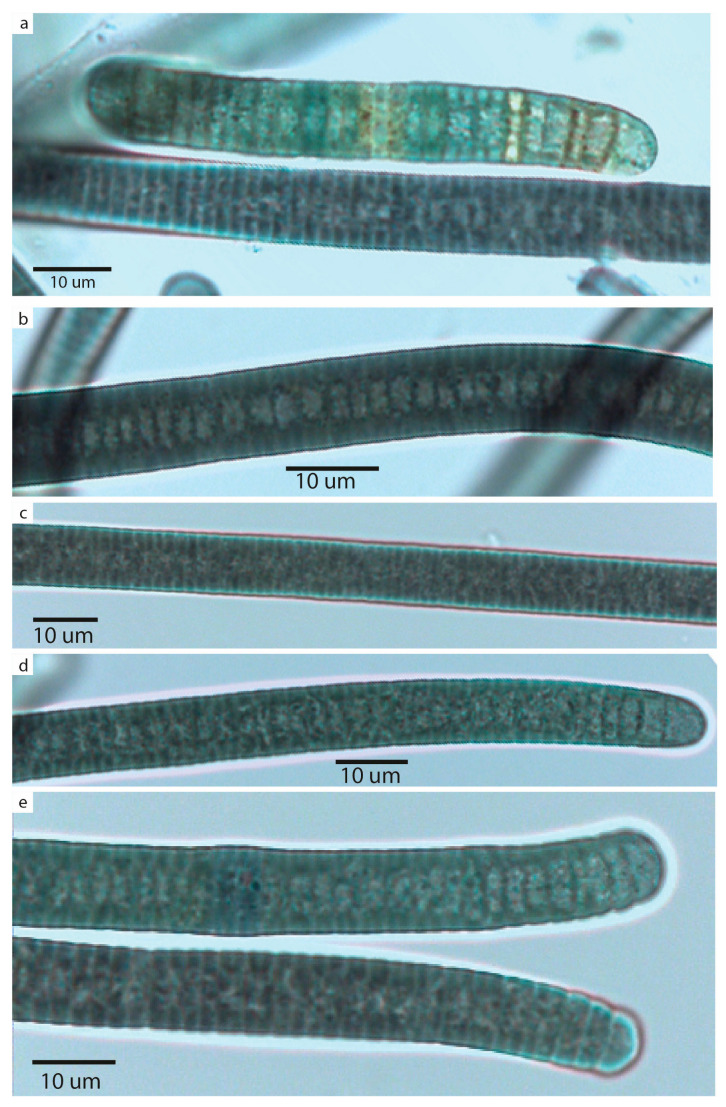
*Reticulonema bolivianum* gen. et sp. nov. (**a**) Trichome and hormogonium. (**b**–**e**) Details of different parts of the trichomes and apices. The gas vesicles are visible in figures (**d**,**e**).

Figure 3 and Figure 4

In culture, thallus formed a net-like structure attached to the flask walls. Trichomes were entangled, attenuated toward the ends, slightly constricted, and motile. Trichome ends were attenuated and commonly bent, with conical or rounded apical cells. Sheaths were absent. Cells were discoid, 2.5–3 um long, and 10–14 um wide with vacuole-like structure in the center. Cell content was dark-green, with small granules and gas vesicles. Necridia and hormogonia were present.

Etymology: “*Reticulum”* is the Latin word for “net”. “*bolivianum”* concerns the origin of the new taxon.

Holotype: Collected from the margin of the brackish–alkaline lake Pastos Grandes, Bolívia (21°36′17.47″ S 67°51′00.37″ W), in 2024 by Vitor M. Vasconcelos. Deposited in the University of Porto herbarium under the code PO-T5171.

Type strain: LEGE 231229 (PQ314729).

Habitat: Laguna Pastos Grandes’ brackish and alkaline lake with borax. pH 8.1. Water salinity 0.23 ppt (0.023%).

## 3. Discussion

For the description of *Reticulonema bolivianum* gen. et sp. nov. and the assessment of the current phylogenetic status of the genus *Capilliphycus*, we built three phylogenies using three different methods based on ML and BI algorithms. All the constructed trees were in agreement regarding the phylogenetic position of the studied genera. Moreover, the 16S rRNA gene identities and morphological and ecological analyses confirmed the phylogenetic findings.

In all the phylogenies, *Reticulonema bolivianum* gen. et sp. nov. was positioned within the Microcoleaceae cluster, with strong phylogenetic support. Its clade was distinct from any other cyanobacterial genera. Moreover, the low 16S rRNA gene identity values with the closest phylogenetic relatives, reaching a maximum of only 92.5% with *Okeania*, left no doubt that *Reticulonema bolivianum* gen. et sp. nov. was a distinct cyanobacterial taxon. It is known that 16S rRNA gene identity values below 94.5% between two strains strongly indicate that they belong to different genera [23].

Additionally, the morphological and ecological analyses were in agreement with the phylogenetic and identity findings. *Reticulonema bolivianum* gen. et sp. nov. differed from *Dapis, Tenebriella*, and *Okeania*, the closest phylogenetic relatives, primarily by the presence of aerotopes, which are absent in the latter three genera. Moreover, *Reticulonema bolivianum* gen. et sp. nov. differed from *Dapis* by the attenuation of the trichomes and the lack of mucilaginous sheaths. *Dapis* does not exhibit attenuation and has thin sheaths enclosing the trichomes. Regarding habitats, the mew genus was the only one within this cluster, which was sampled from brackish and alkaline environments. Further evidence supporting the new genus includes previous phylogenetic and 16S rRNA identity analyses showing that PMC strains in the *Reticulonema* clade are unrelated to any known cyanobacterial genus [24]. Our study is aligned with these results.

The case of *Capilliphycus* is much more complicated. The genus *Capilliphycus* is morphologically identical to *Lyngbya* and phylogenetically close related to *Lyngbya*-like genera such as *Sirenicapillaria* Berthold et al., *Affixifilum* Lefler et al.*, Neolyngbya* Caires et al.*, Limnoraphis* Komárek et al., and *Tigrinifilum* Berthold et al. [15,20,21,22]. These genera were formerly designated within the “Sirenicapillariaceae” family [15] but currently are considered to be under the family Microcoleaceae circumscription [25].

Originally, *Capilliphycus* was denoted as *Capillus* Caires et al. and then officially validated as *Capilliphycus* [14]. Upon the initial description of *Capilliphycus*, two species were characterized: *C. salinus* and *C. tropicalis.* After the original description of the genus, the work of Berthold et al. [15] was the only one showing *Capilliphycus* with an “apparent” monophyletic status. In this paper, although the BI phylogeny showed the genus to be monophyletic, there was no statistical support for the ML analysis, meaning that in the ML tree, the genus was polyphyletic. The phylogeny of Lefler et al. [21], in which *Affixifilum* was described, also aligns with our findings, demonstrating the polyphyletic nature of *Capilliphycus*. In their study, *C. salinus* and *C. tropicalis* were phylogenetically positioned in distinct clades. Hentschke et al. [26], in a revision of newly described genera from Brazil, also show the genus to be polyphyletic. The work of Caires et al. [13] showed a monophyletic *Capilliphycus* in the original description of the genus, but at that time the authors did not include *Neolyngbya*, *Sirenicapillaria*, and *Affixifilum* in their phylogeny because they had not yet been described.

As anticipated, in both our first-round phylogeny (Figure 1) and in the second-round BI tree (Figure 2), *Capilliphycus* was polyphyletic. In the ML phylogeny, the genus was monophyletic; however, it presented no bootstrap support (ML = 57). It is important to report that the cluster encompassing *Capilliphycus* sensu lato, *Sirenicapillaria*, and *Limnoraphis* was consistent and exhibited strong phylogenetic support in all phylogenies, indicating a close phylogenetic relationship among these genera.

The 16S rRNA gene identity among “Sirenicapillariaceae” genera was remarkably high and already reported [15]. This work found a mean value of 97.2% within this family. Our results are in agreement with that outcome, considering that we found a mean of 96.4% (±1.5). We also found that morphologically very different genera, such as *Limnoraphis* and *Limnospira*, exhibited high identity values, reaching 95.5%.

Regarding morphology, it was not possible to establish markers for each of the “Sirenicapillariaceae” genera (Table 4).

Based on these findings, two possible hypotheses arise: (1) *Capilliphycus* must be separated in two distinct genera, or (2) all “Sirenicapillariaceae” must be merged into a single genus.

Considering the 16S rRNA gene phylogenetic and identity analyses, if we follow the first option, we would have to separate *Capilliphycus* I and “*Capilliphycus*” II clades, which share 98.4% 16S rRNA gene identity, as different genera. This value is very close to the species threshold [23], which considers that strains with more than 98.7% identity should be included in the same species. The second option would lead to the combination of *Limnospira*, a non-*Lyngbya*-like genus, with morphologically and ecologically very distinct genera, such as *Tigrinifilum*, *Affixifilum*, *Neolyngbya*, *Sirenicapillaria*, *Limnoraphis*, and *Capilliphycus*. We are equally not comfortable in doing this. At this moment, we cannot solve this paradox, and further studies must be conducted to clarify the phylogeny of this group.

The 16S–23S ITS secondary structures of *Capilliphycus*, *Limnoraphis*, *Sirenicapillaria*, and *Tigrinifilum* are shown in Berthold et al. [15]. While these structures exhibit some differences among the genera, they are remarkably similar in terms of structure, sequence, and length. The determination of how distinct these structures must be to justify the separation of genera remains a subjective decision. Currently, there is no statistical method available to compare these structures, making them insufficient as a decisive factor for distinguishing cyanobacterial taxa. These findings further emphasize the close taxonomic relationship among the genera of the “Sirenicapillariaceae” family. In the case of *Reticulonema bolivianum* gen. et sp. nov., the use of 16S–23S ITS secondary structures was unnecessary due to its distinct phylogenetic position and the very low 16S rRNA gene identity that it shares with any other cyanobacterial genus.

Another important point that must be discussed in the Microcoleaceae phylogeny is the uncertainty of a reference strain for *Lyngbya* Gomont. Some strains labeled as the type species *L. confervoides* are sequenced and available in GenBank (NCBI), but no detailed morphological and ecological analyses were conducted for these strains, making the genus not yet validated by molecular analysis. There is no formal designation of a reference strain for *Lyngbya*, such as made for *Oscillatoria* Gomont [27]. Considering that, we emphasize that a revision of *Lyngbya* is needed. Currently, there are many “*Lyngbya*” sequences in the National Center for Biotechnology Information (NCBI) that are misidentified and positioned in different clades across Cyanobacteria phylogenies.

In conclusion, our paper presents a report of a new cyanobacterial genus from a Bolivian extreme environment. These habitats are currently poorly studied and have already been proved to be a valuable source of new taxonomical discoveries. Beyond the description of the new taxon, it is also possible to affirm that further studies are needed to elucidate the phylogenetic relationships among Microcoleaceae genera due to the high 16S rRNA gene similarities among them. Approaches including not only the 16S rRNA gene but also phylogenomics, morphology, ecology, and ultrastructure might help to solve the incongruences and make the taxonomic analyses more accurate.

## 4. Materials and Methods

### 4.1. Sampling and Morphological Analysis

A cyanobacterial mat was collected from the margin of the brackish–alkaline lake Pastos Grandes, Bolivia (21°36′17.47″ S 67°51′00.37″ W). After sampling, the biomass was immediately enriched with Z8 liquid medium [28] in a Falcon tube and therefore transported to the laboratory. In the field, the measured pH was 8.1, and the salinity was 0.23 ppt (0.023%).

From this sample, the strain LEGE 231229 was manually isolated by using a tapered Pasteur pipette (VWR International GmbH, Darmstadt, Germany) to pick a single filament, which was then inoculated in a flask containing Z8 liquid medium. The strain is currently maintained in Z8 liquid medium [28] under the following conditions: 19 °C, 12:12 h light/dark cycle (25 μmol photons m^−2^ s^−1^). The strain was microphotographed, recorded, and analyzed by the LEICA LAS version 4.12.0 image analysis software (Leica Microsystems Limited and CMS GmbH, Mannheim, Germany). The measurements were performed in 30 different cells and were carried out at various positions of the slide preparation. From this culture, an aliquot was lyophilized and preserved in the University of Porto Herbarium under the code PO-T5171.

### 4.2. DNA Extraction, PCR Amplification and Sequencing

The cyanobacterial filaments from LEGE 231229 were harvested from the culture, and the total genomic DNA (gDNA) of the strain was extracted using the NZY Microbial gDNA Isolation Kit (NzyTech, Lisbon, Porugal), according to the manufacturer’s protocol. Specific cyanobacteria primers were used for gene amplification, including primers 27SF and 1494R [29].

The PCR reactions were performed in a Veriti Thermal Cycler (Veriti 9902, Applied Biosystems, Thermo Fisher Scientific, Waltham, MA, USA). The final reaction volume was 20 μL, consisting of 5.9 μL of molecular biology-grade water, 4 μL of Green GoTaq Flexi Buffer, 2 μL of MgCl_2_, 2 μL of each forward and reverse primer, 1.5 μL of deoxynucleotide triphosphate (dNTPs), 0.5 μL of bovine serum albumin (BSA), 0.1 μL of GoTaq Flexi DNA Polymerase (Promega, Madison, WI, USA), and 2 μL of genomic DNA [30]. The 16S rRNA gene sequence was obtained upon PCR amplification using the following conditions: an initial denaturation step at 94 °C for 4 min, followed by 30 cyc1es of DNA denaturation at 94 °C for 20 s, primer annealing at 50 °C for 30 s, strand extension at 72 °C for 2 min, and a final extension step at 72 °C for 7 min.

The PCR products were separated using 1% (*w*/*v*) agarose gel stained with SYBR Safe DNA gel stain (Invitrogen by Thermo Fisher Scientific, Waltham, MA, USA), and a DNA fragment of the expected size was excised from the gel and purified using the NZYGelpure kit (Nzytech, Lisbon, Portugal), following the manufacturer’s instructions. Finally, the purified fragment was sent for sequencing (separately) with the primers 359F, 781R [31], 27SF, 1494R [29], and 1114F [32]. Sequencing was performed by Sanger dideoxy sequencing at GATC Biotech (Ebersberg, Germany), and the nucleotide sequences obtained were manually inspected for quality and assembled using Geneious Prime 2023.2.1 software (Biomatters Ltd., Auckland, New Zealand). Before phylogenetic analysis, the sequences were checked for possible chimera formation using DECIPHER software 2.27.2 [33]. To assess the presence and quality of the DNA obtained from extraction and PCR, we performed electrophoresis on a 1% (*w*/*v*) agarose gel stained with SYBR Safe DNA gel stain (Invitrogen by Thermo Fisher Scientific, Waltham, MA, USA). The confirmation of high-molecular-weight DNA was based on the presence of a clear band observed in the gel. The sequence was deposited in GenBank (National Center for Biotechnology Information, NCBI) under the ID PQ314729.

### 4.3. Phylogenetic Analysis

The phylogenetic analyses were conducted in two rounds. To position LEGE 231229 among the cyanobacterial families, in the first round, we aligned our 16S rRNA gene sequence with those of 400 cyanobacterial reference strains. The final alignment contained 826 informative sites. The phylogenetic reconstruction was conducted using the FastTree method [34], with a bootstrap value set by default to 1000, according to the manual. The command used to run the phylogeny was “FastTree-gtr-nt alignment_file > tree_file”. The tree was edited using iTOL [35].

For the second round, we selected the cyanobacterial sequences phylogenetically more closely related to our strain, such as the Microcoleaceae genera [25], and other sequences retrieved from GenBank (NCBI) by BLAST, resulting in 98 sequences. The final alignment contained 940 informative sites. Then, the phylogenetic trees were built using maximum likelihood (ML) and Bayesian Inference (BI) analyses. GTR+G+I evolutionary model was selected by MEGA11: Molecular Evolutionary Genetics Analysis version 11 [36]. The robustness of ML tree was estimated by bootstrap percentages, using 1000 replications using IQ-Tree online version v1.6.12 [37]. The Bayesian tree was constructed in two independent runs, with four chains each, for 5 × 10^6^ generations, and the burning fraction was set to 0.25, with a sample frequency 1000, using MrBayes [38] in Cipres Gateway [39]. The processing and visualization of these trees were carried out using FigTree v1.4.4 (http://tree.bio.ed.ac.uk/software/figtree/ (accessed on 1 August 2024)).

For all analyses, the sequences were aligned using MAFFT [40], and the outgroup used was *Gloeobacter violaceus* PCC 8105 (AF132791). An 16S rRNA gene identity (p-distance) matrix was generated using MEGA11.

The review of the phylogenetic status of the genus *Capilliphycus* was performed in the same phylogenies cited above. To compose the *Capilliphycus* dataset of sequences, we retrieved the *Capilliphycus* 16S rRNA gene sequences from [13], where the genus was originally described. We then added the reference strains from [15], which included *C. flaviceps* and *C. guerandensis*. Finally, we included all *Capilliphycus* sequences with more than 1000 nucleotides that resulted from an NCBI search for “Capilliphycus 16S”.

## Figures and Tables

**Table 1:** *p*-distance (percentage of genetic identity) of *Reticulonema bolivianum* gen. sp. nov. and the phylogenetic closest related genera.

		1	2	3	4
1	*Reticulonema* (5 strains)	97.8–100			
2	*Dapis* (2 strains)	89.8–90.9	99.6		
3	*Tenebriella* (1 strain)	90.2–91.1	95.9–96	#	
4	*Okeania* (3 strains)	91.2–92.5	96.1–96.4	97.2–97.4	99.7–99.9

# = only one strain analyzed.

**Table 2:** Morphological characteristics and habitats of *Reticulonema bolivianum* gen. et sp. nov. and phylogenetically closest related genera.

	*Reticulonema bolivianum* gen. et sp. Nov.	*Dapis* [16]	*Tenebriella* [17]	*Okeania* [18]
Thallus	Entangled trichomes	Entangled filaments	Forming mats	Not described
Trichomes	Attenuated toward the ends, slightly constricted. Trichome ends attenuated, commonly bent	Cylindrical, not attenuated toward the ends. Not or slightly constricted	Slightly attenuated toward the ends	Cylindrical or attenuated toward the ends. Constricted or not
Motility	Yes	Not described	Yes	Not described
Apical cell	Conical or rounded	Cupuliform or rounded	Rounded. Facultatively with calyptra	Rounded
Sheaths	Absent	Thin	Facultative. Sometimes lamellate	Present
Cells	Discoid	Discoid	Discoid	Discoid
Cells measurements (um)	2.5–3 long × 10–14 wide	(1) 2–3 (4) long × (28) 30–34 (40) wide	1.5–5 long × 9–20 wide	(1) 2–4 (5) long × (9) 10–45 (50) wide
Cell content	Dark-green, with small granules and gas vesicles	Granulose	Pale greyish green to golden brown and purple	Not described
Aerotopes	Present	Present	Absent	Absent
Habitat	Mat at the margin of a brackish–alkaline lake with borax. pH 8.1. Water salinity 0.23 ppt (0.023%)	Marine. 2–30 m deep	Mainly freshwater or terrestrial	Marine. Coral reefs, 0.5–30 m deep

**Table 3:** *p*-distance (percentage of genetic identity) among “*Sirenicapillariaceae”* taxa based on 16S rRNA gene sequences.

		1	2	3	4	5	6	7	8
1	*Neolyngbya*	96.9–100							
2	*Affixifilum*	95.6–96.4	99.9–100						
3	*Sirenicapillaria*	94.8–96.1	95.3–96.2	98.6–100					
4	*“Capilliphycus”* II	94.6–96.4	95.3–95.9	96–97	99.1–100				
5	*Capilliphycus* I	93.2–96.2	93.1–96.1	94.1–97	95.6–98.4	96.4–100			
6	*Limnoraphis*	95–96	95.3–95.5	96.5–97.3	97.5–98.1	95.6–98.3	99.9–100		
7	*Limnospira*	95–96	94.6–94.8	94.3–95	94.2–95.1	93.4–96	95.3–95.5	99.9–100	
8	*Tigrinifilum*	94–96	94.3–95	94.1–95.2	94–95.5	92–96	94.7–95	95.4–96.5	98.4–99.5

**Table 4:** Morphological characteristics and habitats of *Capilliphycus* and phylogenetically closest related genera.

Taxa	*Limnoraphis* [19]	*Lyngbya confervoides* [20]	*Sirenicapillaria* [15]	*Tigrinifilum* [15]	*Affixifilum* [21]	*Neolyngbya* [22]	*Capilliphycus* I [13]	*Capilliphycus* II [13]
Filament morphology	Straight or slightly curved	Straight or entangled at the base, later erect	Straight	Straight	Straight or waved	Straight	Straight or flexuous, rarely coiled	Straight, sometimes coiled
Filament arrangement	Solitary, free-floating or small aggregations	Fasciculate, forming mats	Entangled	Entangled, forming mats	Entangled, forming mats	Entangled, forming mats	Fasciculate, forming mats	Fasciculate or entangled, forming mats
Sheaths	Obligatory, firm, think or thick, hyaline	Obligatory, firm, think or thick, hyaline, lamellated (older)	Obligatory, thin or thick, sometimes lamellated	Facultative	Obligatory, thin or thick, hyaline	Obligatory, thin or thick, hyaline, sometimes lamellated	Facultative, firm, thin or thick, hyaline	Facultative, firm, thick, lamellated or not, hyaline
Trichomes	Cylindrical, not or slightly constricted	Cylindrical, not constricted	Slightly attenuated	Constricted or not	Attenuated, slightly constricted	Cylindrical, slightly constricted or not	Cylindrical, sometimes attenuated. Slightly constricted	Cylindrical, constricted
Cells	Discoid	Discoid	Discoid	Discoid	Discoid	Discoid	Discoid	Discoid
Cell content	Not described	Homogenous or granulose	Heavily pigmented	Homogenous	Granulose	Granulose	Homogenous or granulose	Homogenous or granulose
Aerotopes	Facultative	Absent	Absent	Absent	Present	Present	Present	Present
Apical cell	Without thickening	Rounded, without thickening in outer membrane	Rounded or conical	Rounded or conical	Rounded, commonly with calyptra	Rounded or conical, rarely thickened	Rounded, conical, without thickening	Rounded or conical. Rarely with thickening in outer membrane
Filament width (μm)	5–25	12–30	12.5–94	9–19	8.1–29.8	7.8–24.7	14–29.2	12–18.9
Habitat	Freshwater	Marine, on rocks	Marine, benthic	Marine or hypersaline lakes, benthic or planktonic	Marine, on rocks or sand soils	Marine, on rocks or sand soils	Marine, on rocks. Hypersaline pools	Marine, on rocks or floating mats
Thylakoids arrangement	Unknown	Unknown	Unknown	Unknown	Unknown	Irregular	Parietal or irregular	Irregular

## Data Availability

Data are contained within the article.
